# A Marine Bivalve (*Entodesma navicula*) Extract Suppresses the Replication of Influenza Viruses and Flaviviruses in Association with Reduced MLKL Phosphorylation

**DOI:** 10.3390/ani16203226

**Published:** 2026-10-14

**Authors:** Hyeon Jeong Moon, Gun Lee, Seok-Chun Ko, Jeong Min Lee, Ji-Yul Kim, Mi-Jin Yim, Jaewoo Choi, Chung-Do Lee, Jun Bong Lee, Jun-Gyu Park, Yeong-Bin Baek, Sang-Ik Park

**Affiliations:** 1 Department of Veterinary Pathology, Chonnam National University, Gwangju 61186, Republic of Korea; dals404@naver.com (H.J.M.); e05311cht@naver.com (J.C.); 2 Department of Veterinary Pathology, College of Veterinary Medicine and BK21 FOUR Program, Chonnam National University, Gwangju 61186, Republic of Korea; udlrjs77@naver.com (G.L.); cndehsla@gmail.com (C.-D.L.); 3 Department of Biomaterial Research, National Marine Biodiversity Institute of Korea, Seocheon 33662, Republic of Korea; seokchunk@mabik.re.kr (S.-C.K.); lshjm@mabik.re.kr (J.M.L.); jiyul2224@mabik.re.kr (J.-Y.K.); mjyim@mabik.re.kr (M.-J.Y.); 4 Department of Food and Environmental Hygiene, College of Veterinary Medicine, Chonnam National University, Gwangju 61186, Republic of Korea; jblee12@jnu.ac.kr; 5 Department of Veterinary Zoonotic Diseases, College of Veterinary Medicine, Chonnam National University, Gwangju 61186, Republic of Korea; kingsalt@jnu.ac.kr; 6 Animal Medical Institute, Chonnam National University, Gwangju 61186, Republic of Korea

**Keywords:** *Entodesma navicula*, marine bivalve, influenza virus, Zika virus, dengue virus, necroptosis, MLKL phosphorylation, post-infection antiviral activity

## Abstract

Emerging influenza viruses and mosquito-borne flaviviruses continue to challenge animal and public health, highlighting the need for antivirals that remain effective after infection. This study evaluated the in vitro activity of a 70% ethanolic whole-specimen extract of the marine bivalve *Entodesma navicula* (ENE) against influenza A/Puerto Rico/8/1934 (H1N1; A/PR8), A/Wisconsin/15/2009 (H3N2), influenza B/Florida/78/2015, Zika virus, and dengue virus serotype 2. Antiviral activity was assessed using time-of-addition analysis, viability-based cytopathic-effect assays, reverse-transcription quantitative PCR, infectious virus titration, immunofluorescence, Western blotting, and flow cytometry. In the A/PR8 model, pre- and co-treatment produced no consistent inhibition. By contrast, post-infection ENE suppressed all tested viruses. For A/PR8, Zika virus, and dengue virus, this activity was supported by reduced viral genome abundance, infectious progeny, and viral antigen expression. In A/PR8-infected A549 cells, ENE reduced phosphorylation of receptor-interacting serine/threonine-protein kinase 3 (RIPK3) and mixed lineage kinase domain-like pseudokinase (MLKL). ENE also reduced the proportion of cells positive for phosphorylated MLKL within Zika- and dengue-virus-infected Vero E6 populations. These findings identify *E. navicula* as an underexplored source of post-infection antiviral bioactivity associated with attenuated necroptosis-related signaling. Isolation of active constituents and causal validation in primary cells and animal models are needed.

## Simple Summary

Viruses such as influenza, Zika, and dengue threaten animal and human health, and new treatments are needed because these viruses can change rapidly and spread between species or through mosquitoes. Marine animals are an underused source of natural compounds that may help control viral infections. We tested an extract from *Entodesma navicula*, a marine shellfish, against several influenza viruses, Zika virus, and dengue virus in laboratory-grown cells. The extract was effective when applied after infection and reduced viral genetic material, the production of infectious virus particles, and viral proteins. It also lowered the activation of proteins linked to necroptosis, an inflammatory type of programmed cell death that can occur during viral infection. The active substances in the extract are not yet known, and the study was limited to cell-based experiments. However, the findings show that this little-studied marine animal may contain useful antiviral compounds. Identifying these compounds and testing them in animal models could support the development of new antiviral approaches for animal health and diseases shared between animals and people.

## 1. Introduction

Emerging and re-emerging RNA viruses continue to challenge animal health, public health, and One Health preparedness because they combine rapid genetic change with extensive dependence on host-cell processes. Influenza A virus (IAV) is a prototypical example: its segmented genome supports continuous antigenic evolution and reassortment, facilitating adaptation among avian and mammalian hosts and complicating durable antiviral control [1,2]. Influenza B virus (IBV), although distinct from IAV in its host range and evolutionary ecology, provides a complementary target for evaluating antiviral activity across influenza types [1]. Zika virus (ZIKV) and dengue virus (DENV) occupy mosquito–vertebrate transmission systems rather than respiratory transmission routes, but their emergence similarly illustrates the convergence of viral evolution, vector ecology, and vertebrate susceptibility [3]. Together, these biologically diverse viruses support the search for post-infection interventions that act on conserved viral or host processes and retain activity across multiple viral strains or families.

Among conserved host processes, regulated cell death is a critical determinant of viral replication, tissue injury, and antiviral immunity. Necroptosis is a regulated lytic cell-death pathway principally mediated by receptor-interacting serine/threonine-protein kinase 3 (RIPK3) and mixed lineage kinase domain-like pseudokinase (MLKL) [4]. Following its phosphorylation by RIPK3, activated MLKL oligomerizes and translocates to cellular membranes, resulting in membrane permeabilization and necrotic cell death [4,5]. Although this response can eliminate infected cells before completion of the viral replication cycle, membrane disruption can release damage-associated molecular patterns and amplify inflammatory tissue injury [6]. Accordingly, MLKL phosphorylation represents a central activation step in necroptotic signaling, while its biological consequences depend on the virus, host cell, infection stage, and magnitude of pathway activation.

Interactions between RNA viruses and necroptotic signaling are highly context-dependent. During IAV infection, Z-DNA-binding protein 1 (ZBP1) recognizes influenza viral ribonucleoprotein-associated RNA and virus-derived Z-RNA structures, subsequently engaging RIPK3-dependent cell-death pathways that include MLKL-mediated necroptosis [7,8,9,10]. Genetic studies indicate that these pathways can restrict viral dissemination and contribute to host protection. ZIKV likewise induces RIPK3-dependent MLKL phosphorylation and necroptosis in human astrocytes, where inhibition of RIPK3 increases viral replication, suggesting an antiviral function in this specific cellular context [11]. Conversely, DENV has been reported to target RIPK1 and suppress induced MLKL phosphorylation and inflammatory signaling in myeloid cells [12]. These divergent observations demonstrate that necroptosis is neither uniformly antiviral nor uniformly pathogenic. Changes in MLKL phosphorylation should therefore be interpreted together with viral burden, infection stage, and cellular context.

Marine invertebrates represent a chemically and immunologically distinct source of potential antiviral molecules. Bivalves are continuously exposed to microorganisms and viruses in aquatic environments and rely predominantly on evolutionarily conserved innate defense systems, including soluble hemolymph factors, lectins, antimicrobial peptides, and multifunctional immune proteins [13]. Crude extracts derived from marine bivalves have previously shown activity against mammalian viruses, including influenza viruses [14], and broader screening studies have examined antiviral fractions from several bivalve and gastropod species [15]. Antiviral activity has also been demonstrated in Pacific oyster hemolymph and its major hemolymph protein, cavortin, as well as in the mussel-derived peptide myticin C [16,17,18]. Collectively, these findings establish marine bivalves as plausible sources of antiviral bioactivity. Nevertheless, previous studies have concentrated mainly on a limited number of oyster and mussel species and have frequently employed herpesvirus-centered models. The ability of taxonomically distinct bivalves to inhibit respiratory and vector-borne RNA viruses, particularly in association with regulated host-cell-death pathways, remains insufficiently characterized.

Although *Entodesma navicula* has been examined from morphological and biomineralization perspectives, including the organization and formation of its granular prismatic shell microstructure, its biological and pharmacological properties remain largely uncharacterized [19]. Evidence from other marine bivalves nevertheless supports this group as a source of antiviral molecules. Extracts from mussels, oysters, and clams, including *Perna viridis*, *Crassostrea madrasensis*, and *Meretrix casta*, have shown inhibitory activity against influenza A and B viruses [14]. Acidic extracts from *Cerastoderma edule*, *Ruditapes philippinarum*, and *Ostrea edulis* have also demonstrated antiviral activity against herpes simplex virus type 1 in cell-based assays [15]. In addition, pyropheophorbide a isolated from the mussel *Musculus senhousei* exhibited antiviral activity against multiple influenza A virus strains, illustrating that defined antiviral constituents can be recovered from bivalve-derived materials [20]. These findings provide a rationale for investigating previously uncharacterized bivalve species, including *E. navicula*, as potential sources of antiviral bioactivity.

Accordingly, this study evaluated the post-infection antiviral activity of an *E. navicula* extract (ENE) against representative influenza A and B viruses and flaviviruses and examined whether this activity was associated with altered RIPK3–MLKL signaling. The study was designed to expand marine-animal antiviral bioprospecting and provide a basis for subsequent identification and mechanistic validation of the active constituents.

## 2. Materials and Methods

### 2.1. Marine Bivalve Extract and Resource Information

An extract prepared from the marine bivalve *Entodesma navicula* was supplied by the Microbial Marine BioBank (MMBB) of the National Marine Biodiversity Institute of Korea (MABIK), Republic of Korea, under resource no. MABIK NP20250067. According to the accompanying resource information, the specimen was collected from the East Sea off Goseong-gun, Gangwon-do, Republic of Korea, and taxonomically identified as *E. navicula* by MABIK on the basis of morphological characteristics. Whole-specimen material was extracted with 70% ethanol using an ultrasound-assisted extraction procedure before distribution. For consistency throughout this study, the supplied material is hereafter referred to as the ENE.

For cell-based experiments, ENE was dissolved in dimethyl sulfoxide (DMSO) at a stock concentration of 100 mg/mL and diluted in the appropriate assay medium immediately before use. The final DMSO concentration did not exceed 1% (*v*/*v*). Vehicle controls were prepared using the DMSO concentration applied in the corresponding experiment.

### 2.2. Cell Lines and Viruses

Madin–Darby canine kidney cells (MDCK; ATCC CCL-34), African green monkey kidney cells (Vero E6; ATCC CRL-1586), and human lung adenocarcinoma epithelial cells (A549; ATCC CCL-185) were obtained from the American Type Culture Collection (ATCC, Manassas, VA, USA). Cells were cultured in Dulbecco’s Modified Eagle Medium (DMEM) supplemented with 10% fetal bovine serum, 100 U/mL penicillin, and 100 μg/mL streptomycin. All cultures were maintained at 37 °C in a humidified atmosphere containing 5% CO_2_.

The influenza viruses used in this study were influenza A/Puerto Rico/8/1934 (H1N1; A/PR8), influenza A/Wisconsin/15/2009 (H3N2; A/Wisconsin), and influenza B/Florida/78/2015 virus of the Victoria lineage (B/Florida). ZIKV strain PRVABC59, belonging to the Asian/American lineage, and dengue virus serotype 2 (DENV2) were obtained from the National Culture Collection for Pathogens (Cheongju, Republic of Korea).

Influenza virus stocks were propagated in MDCK cells using serum-free DMEM supplemented with 1 μg/mL TPCK-treated trypsin. All preliminary antiviral screening, time-of-addition, and confirmatory ENE-treatment experiments involving influenza viruses were performed in A549 cells. ZIKV and DENV2 were amplified and assayed in Vero E6 cells using DMEM without TPCK-treated trypsin. Thus, MDCK cells were used for influenza virus propagation, whereas A549 and Vero E6 cells served as the experimental infection models for influenza viruses and flaviviruses, respectively. Infectious virus titers were determined using the 50% tissue culture infectious dose (TCID_50_) assay as described below.

### 2.3. Cell Viability and Cytotoxicity Assessment

A549 or Vero E6 cells were seeded into 96-well culture plates at approximately 5.0 × 10^4^ cells per well and incubated for 24 h. ENE was prepared as a two-fold serial dilution over a concentration range of 3.9–1000 μg/mL. To determine cytotoxicity under conditions corresponding to the preliminary antiviral screening, uninfected cells were exposed to each ENE concentration for 1 h. The treatment medium was then removed, and the cells were subjected to a 1 h mock-adsorption step under the corresponding infection-medium conditions. The same concentration of ENE was subsequently reapplied and maintained until the 48 h endpoint. Vehicle-treated controls received the corresponding concentration of DMSO according to the same exposure schedule. Chloroquine-associated cytotoxicity was evaluated using the same combined pre- and post-exposure protocol.

Cell viability was determined using the MTT (Sigma-Aldrich, St. Louis, MO, USA) reduction assay. To mitigate potential direct colorimetric interference by the crude extract, the ENE-containing medium was aspirated, and the cell monolayers were washed with phosphate-buffered saline before adding the MTT reagent. Cell-free blank wells were also utilized for background subtraction. Following incubation with MTT reagent, the resulting formazan crystals were solubilized, and absorbance was measured using a microplate reader at 540 nm. Cell viability was calculated relative to that of the corresponding vehicle-treated control. The concentration that reduced cell viability by 50% was defined as the half-maximal cytotoxic concentration (CC_50_) and was estimated using nonlinear regression analysis.

### 2.4. Preliminary Antiviral Screening Using Combined Pre- and Post-Infection Treatment

To establish preliminary concentration–response profiles and determine antiviral selectivity, a combined pre- and post-infection treatment protocol was used as an initial broad screening step to maximize the probability of detecting potential antiviral activity and to establish the baseline half-maximal inhibitory concentration (IC_50_) and selectivity index (SI) values, with modifications of previously described methods [21,22,23]. A549 cells were used for A/PR8, A/Wisconsin, and B/Florida infection, whereas Vero E6 cells were used for ZIKV and DENV2 infection.

Cells were pre-treated for 1 h with serially diluted ENE at 1000, 500, 250, 125, 62.5, 31.3, 15.6, 7.8, 3.9, or 0 μg/mL. Following pre-treatment, the ENE-containing medium was removed, and the cells were inoculated with the corresponding virus at a multiplicity of infection (MOI) of 0.1 for 1 h. This specific MOI was empirically selected based on preliminary assay optimizations to establish an optimal analytical window at 48 h post-infection. Higher MOIs lead to rapid single-round replication that can mask antiviral efficacy, while lower MOIs do not produce sufficient viral loads to establish clear statistical differences within the 48-h timeframe. Influenza virus adsorption was performed in infection medium containing 1 μg/mL TPCK-treated trypsin, whereas ZIKV and DENV2 adsorption was conducted in medium without trypsin. After viral adsorption, the inoculum was removed, and the cells were washed with Dulbecco’s phosphate-buffered saline. ENE was then reapplied at the same concentration used during pre-treatment and maintained continuously until 48 h post-infection.

Chloroquine was evaluated as a pharmacological comparator against A/PR8, ZIKV, and DENV2 using the same combined pre- and post-infection treatment schedule. At 48 h post-infection, virus-induced cytopathic effects and cell viability were assessed using the MTT assay. The IC_50_ was estimated from the concentration–response curve for inhibition of virus-induced cytopathic effects.

CC_50_ and IC_50_ values were estimated using four-parameter nonlinear concentration–response regression in GraphPad Prism version 10.5.1 (GraphPad Software, San Diego, CA, USA). The SI was calculated as follows:

$$\mathrm{SI}=\;\frac{{\mathrm{CC}}_{50}}{{\mathrm{IC}}_{50}}$$

The CC_50_, IC_50_, and SI values derived from this assay represent the combined pre- and post-infection screening regimen and not the subsequent post-treatment-only protocol.

### 2.5. Time-of-Addition Experiment and Selection of Confirmatory Post-Treatment Conditions

Time-of-addition experiments were conducted independently of the preliminary antiviral screening using A/PR8 as the representative virus to determine whether ENE acted before, during, or after viral inoculation [24].

For pre-treatment, A549 cells were exposed to ENE for 1 h. The extract was then removed, the cells were washed, and A/PR8 was inoculated at an MOI of 0.1 for 1 h. Following removal of the viral inoculum, the cells were washed and maintained in fresh infection medium without further ENE exposure.

For co-treatment, A/PR8 and ENE were combined immediately before inoculation. The cells were incubated with the virus–ENE mixture for 1 h, after which the inoculum was removed. The monolayers were washed and maintained in fresh infection medium without further ENE exposure.

For post-treatment, the cells were first inoculated with A/PR8 at an MOI of 0.1 for 1 h. After removal of the inoculum and washing, the cells were exposed to ENE. The ENE-containing medium was continuously maintained for the entire 48-h incubation period until sample collection.

Cells and culture supernatants were collected at 48 h post-infection for the assessment of virus-induced cytopathic effects, viral RNA abundance, and infectious progeny production. Unless otherwise indicated, subsequent confirmatory virological and host-response assays were performed using the post-treatment protocol described above. The ENE concentrations used in each assay are specified in the corresponding subsections.

### 2.6. Quantification of Viral RNA

For confirmatory post-treatment experiments, A549 cells infected with A/PR8 were exposed to ENE at 0.01, 0.1, 0.5, 1, 3, 5, 10, 25, 50, or 100 μg/mL for 1 h immediately after viral adsorption. Vero E6 cells infected with ZIKV or DENV2 were similarly exposed to ENE at 0.1, 0.5, 1, 5, 10, 25, 50, 75, or 100 μg/mL. Following the viral adsorption and washing steps, the ENE-containing medium was added and continuously maintained for 48 h until sample collection.

Culture supernatants were collected at 48 h post-infection and clarified to remove cellular debris. Viral RNA was isolated using the QIAamp Viral RNA Mini Kit (QIAGEN, Hilden, Germany) according to the manufacturer’s instructions. One-step reverse-transcription quantitative PCR (RT-qPCR) was performed using the SensiFAST SYBR Lo-ROX One-Step Kit (Bioline Meridian BioScience, Cincinnati, OH, USA).

The following primer pairs were used: IAV PB1 forward, 5′-GGCCCTTCAGTTGTTCATC-3′, and reverse, 5′-GCAGACTTCAGGAATGTG-3′; ZIKV NS5 forward, 5′-GGTCATGATACTGCTGATTGC-3′, and reverse, 5′-CCACTAACGTTCTTTTGCAGAC-3′; and DENV2 prM forward, 5′-AGTTGTTAGTCTRYGTGGACCGAC-3′, and reverse, 5′-TTGCACCAACAGTCAATGTCTTCAGGTTC-3′.

Amplification was performed according to the reagent manufacturer’s recommended cycling protocol. Melting-curve analysis was used to confirm the specificity of the amplification products. Viral RNA levels were normalized to those in the corresponding infected vehicle-control group and expressed as relative log_10_ values.

### 2.7. Infectious Virus Titration

Infectious progeny production was quantified using the TCID_50_ assay [25]. Culture supernatants collected at 48 h post-infection were serially diluted ten-fold in DMEM and inoculated onto confluent monolayers of the appropriate permissive cell line in 96-well plates.

A549 cells were used for the titration of influenza viruses, and the maintenance medium contained 1 μg/mL TPCK-treated trypsin. Vero E6 cells were used for ZIKV and DENV2 titration in medium without trypsin. Plates were incubated at 37 °C under 5% CO_2_, and virus-induced cytopathic effects were evaluated 4 days after inoculation. Infectious titers were calculated using the Reed–Muench method and expressed as log_10_ TCID_50_/mL. Relative changes in viral titers were calculated against the corresponding infected vehicle-control group where indicated.

### 2.8. Immunofluorescence Analysis

A549 cells were seeded in chamber slides and infected with A/PR8 at an MOI of 0.1. Vero E6 cells were similarly prepared and infected with ZIKV or DENV2 at an MOI of 0.1.

Following viral adsorption, the cells were post-treated with ENE as described above and continuously maintained for 48 h. A/PR8-infected cells were treated with 1, 10, or 50 μg/mL ENE, whereas ZIKV- and DENV2-infected cells were treated with 10, 50, or 100 μg/mL ENE.

At 48 h post-infection, cells were fixed with 4% paraformaldehyde for 10 min at room temperature, permeabilized, and blocked with 5% bovine serum albumin for 1 h. Cells were incubated overnight at 4 °C with an antibody against influenza A virus nucleoprotein (NP; Abcam, Cambridge, UK) or the flavivirus group envelope protein antibody 4G2 (Native Antigen Company, Kidlington, UK).

After washing, cells were incubated for 1 h at room temperature with an Alexa Fluor 488-conjugated goat anti-mouse IgG antibody (Thermo Fisher Scientific, Waltham, MA, USA). Nuclei were counterstained with DAPI. Fluorescence images were obtained using an LSM 900 confocal microscope and analyzed using Zen Blue Software 3.1 (Carl Zeiss; Jena, Germany) under identical acquisition settings for all experimental groups.

Viral antigen-associated fluorescence was quantified from randomly selected fields. The number of fluorescence-positive pixels was normalized to the number of DAPI-positive nuclei in each field and expressed as fluorescence intensity per cell. Values were log_10_-transformed before statistical analysis.

### 2.9. Western Blotting

A549 cells were mock-inoculated or infected with A/PR8 at an MOI of 0.1. Following viral adsorption for 1 h, the inoculum was removed, and the cells were washed with phosphate-buffered saline. The cells were subsequently assigned to the following three experimental groups: (1) mock-infected, vehicle-treated controls; (2) A/PR8-infected, vehicle-treated controls; and (3) A/PR8-infected, ENE-treated cultures. ENE was administered at 50 μg/mL and was continuously maintained in the culture medium until sample harvest at 48 h post-infection. The corresponding control cultures received an equivalent volume of vehicle under the same treatment conditions.

At 48 h post-infection, the cells were washed with ice-cold phosphate-buffered saline and lysed in RIPA buffer supplemented with protease and phosphatase inhibitor cocktails. Cell lysates were clarified by centrifugation at 12,000× *g* for 10 min at 4 °C, and protein concentrations were determined using a bicinchoninic acid assay.

Equal amounts of protein from the same biological samples were separated by SDS-polyacrylamide gel electrophoresis and transferred onto nitrocellulose membranes. The membranes were blocked for 1 h at room temperature in Tris-buffered saline containing 0.1% Tween-20 and 5% bovine serum albumin and subsequently incubated overnight at 4 °C with primary antibodies against IAV NP (Abcam), phospho-MLKL at Ser358 (Cell Signaling Technology, Danvers, MA, USA), total MLKL (Abcam, Cambridge, UK), phospho-RIPK3 at Ser227 (Abcam), total RIPK3 (Abcam), or β-actin (Sigma-Aldrich).

After washing, the membranes were incubated for 1 h at room temperature with the appropriate HRP-conjugated secondary antibodies. Immunoreactive bands were visualized using an enhanced chemiluminescence substrate and captured using an iBright CL1500 Imaging System (Thermo Fisher Scientific). Densitometric analysis of the immunoreactive bands was performed using ImageJ software (version 1.54t; National Institutes of Health, Bethesda, MD, USA). IAV NP levels were normalized to β-actin. Phospho-MLKL and phospho-RIPK3 levels were normalized to total MLKL and total RIPK3, respectively, whereas total MLKL and total RIPK3 levels were normalized to β-actin.

### 2.10. Flow Cytometry

Virus-associated MLKL phosphorylation and intracellular viral antigen expression were evaluated by flow cytometry using virus-specific permissive cell models. For the influenza virus experiment, A549 cells were mock-inoculated or infected with A/PR8 at an MOI of 0.1. For the flavivirus experiments, Vero E6 cells were independently mock-inoculated or infected with ZIKV or DENV2 at an MOI of 0.1.

For each virus–cell combination, the cells were assigned to the following three experimental groups: (1) mock-infected, vehicle-treated controls; (2) virus-infected, vehicle-treated controls; and (3) virus-infected, ENE-treated cultures. After viral adsorption for 1 h, the inoculum was removed, and the cells were washed with phosphate-buffered saline. A/PR8-infected A549 cells were treated with 50 μg/mL ENE, whereas ZIKV- or DENV2-infected Vero E6 cells were treated with 100 μg/mL ENE. ENE was applied and continuously maintained for 48 h according to the post-infection treatment protocol described above. The corresponding mock-infected and virus-infected control cultures received an equivalent volume of vehicle under the same treatment conditions. The A/PR8–A549, ZIKV–Vero E6, and DENV2–Vero E6 experimental systems were analyzed independently.

At 48 h post-infection, the cells were harvested and fixed. Following fixation, the cells were permeabilized with 0.1% Triton X-100. For each virus–cell system, a two-color intracellular staining panel was used to simultaneously identify virus-infected cells and evaluate MLKL phosphorylation. Intracellular phospho-MLKL was detected using a rabbit antibody directed against phospho-MLKL at Ser358 (Cell Signaling Technology). Intracellular viral antigens were detected using a mouse antibody against IAV NP (Abcam) in A/PR8-infected A549 cells or a mouse monoclonal antibody against the flavivirus group envelope protein, clone 4G2 (Native Antigen Company), in ZIKV- and DENV2-infected Vero E6 cells. Appropriate fluorophore-conjugated anti-rabbit and anti-mouse IgG secondary antibodies were subsequently applied.

Samples were acquired using an Attune NxT flow cytometer (Thermo Fisher Scientific), and the data were analyzed using Attune NxT software version 3.1.2. Cellular debris was excluded based on forward- and side-scatter characteristics, and cell aggregates were excluded by singlet-cell gating. Fluorescence compensation and positivity thresholds were established using unstained and single-stained controls. Approximately 40,000 singlet-cell events were acquired and analyzed for each sample.

A/PR8-infected cells were identified as NP-positive cells, whereas ZIKV- and DENV2-infected cells were identified as flavivirus envelope protein-positive cells. Intracellular viral antigen expression was assessed by the number and percentage of viral antigen-positive cells. Virus-associated MLKL phosphorylation was evaluated by determining the number and percentage of viral antigen-positive/phospho-MLKL-positive double-positive events. The primary flow cytometric endpoint was expressed as the percentage of phospho-MLKL-positive cells within the corresponding viral antigen-positive population, calculated as follows:

viral antigen+/p-MLKL+ double-positive eventstotal viral antigen+ events×100

### 2.11. Statistical Analysis

All quantitative data were obtained from at least three independent experiments unless otherwise indicated and are presented as the mean ± standard deviation. Statistical analyses were performed using GraphPad Prism software. Comparisons between two groups were performed using Student’s *t*-test, whereas comparisons among multiple groups were analyzed using one-way analysis of variance (ANOVA) followed by Dunnett’s multiple comparisons post hoc test. This specific approach was chosen to properly control the family-wise error rate when comparing multiple experimental ENE treatment concentrations to a single reference group (the virus-infected vehicle control). A *p*-value of less than 0.05 was considered statistically significant.

## 3. Results

### 3.1. Preliminary Screening and Post-Treatment Validation of ENE Against A/PR8

**Figure 1:**
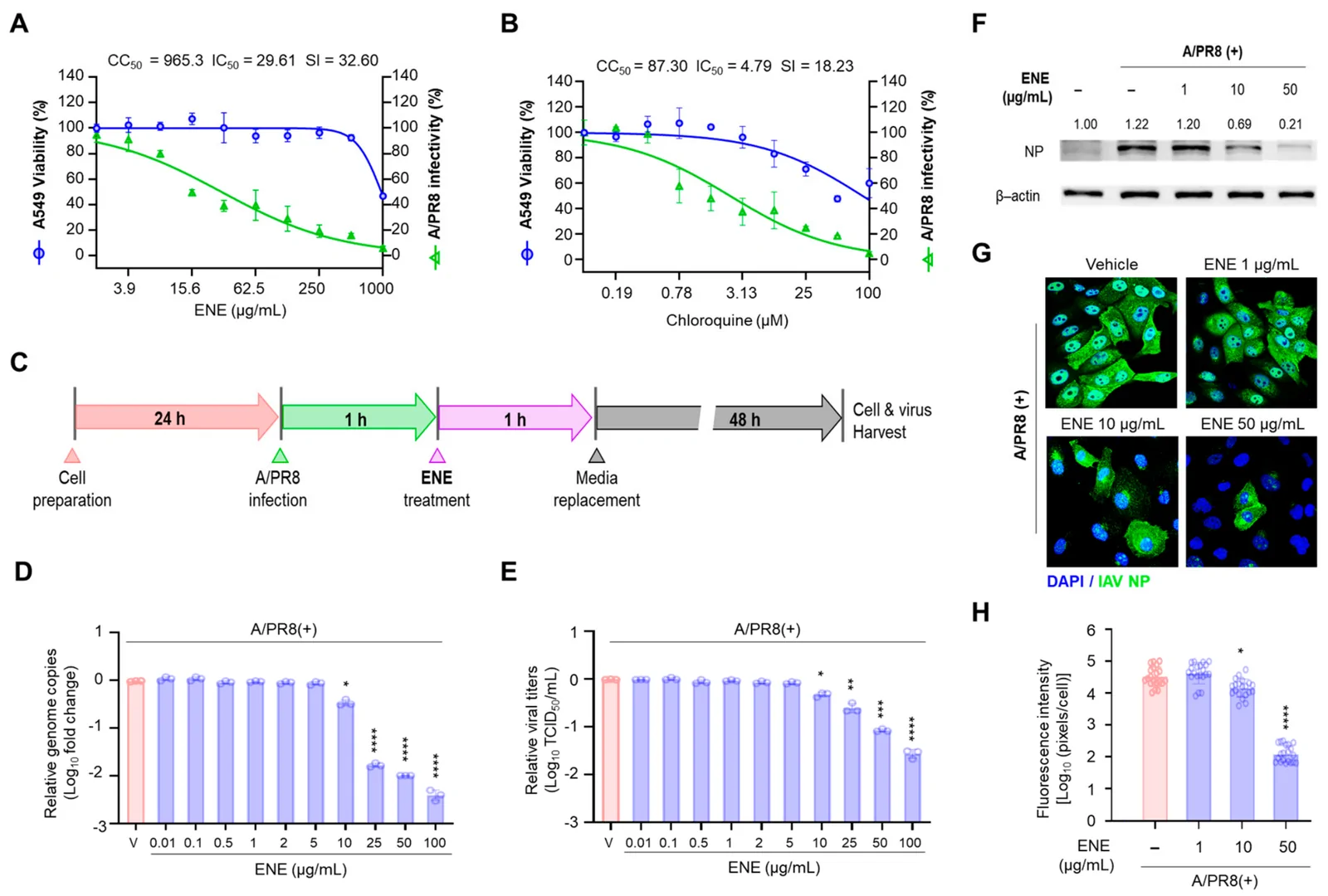
Preliminary concentration–response screening and post-treatment validation of ENE against A/PR8 in A549 cells. (**A**,**B**) Concentration-dependent cytotoxicity and antiviral activity of ENE (**A**) and chloroquine (**B**) under the combined pre- and post-infection screening regimen; the calculated CC_50_, IC_50_, and SI values are shown. (**C**) Schematic representation of the post-treatment-only protocol used for the confirmatory experiments shown in panels (**D**–**H**). (**D**) Relative A/PR8 genome copies (Log_10_ fold change) measured by RT-qPCR following treatment with increasing concentrations of ENE. (**E**) Relative infectious progeny titers (Log_10_ TCID_50_/mL) measured using the 50% tissue culture infectious dose (TCID_50_) assay. (**F**) Western blot analysis of IAV nucleoprotein expression in mock-infected and A/PR8-infected A549 cells treated with vehicle or ENE at 1, 10, or 50 μg/mL. Numbers above the NP bands indicate β-actin-normalized relative intensities. (**G**) Representative immunofluorescence images of IAV NP expression in A/PR8-infected A549 cells treated with vehicle or ENE at 1, 10, or 50 μg/mL. Nuclei were counterstained with DAPI. Scale bar = 20 μm. (**H**) Quantification of NP-associated fluorescence, expressed as log_10_-transformed fluorescence-positive pixels per cell. Quantitative data are presented as the mean ± SD of three independent experiments. *, *p* < 0.05; **, *p* < 0.01; ***, *p* < 0.001; and ****, *p* < 0.0001 versus the A/PR8-infected vehicle-control group. CC_50_, half-maximal cytotoxic concentration; ENE, *Entodesma navicula* extract; IC_50_, half-maximal inhibitory concentration; NP, nucleoprotein; SI, selectivity index; V, virus-infected vehicle-treated control.

A preliminary concentration–response screening assay was first performed using combined pre- and post-infection exposure to ENE over a concentration range of 3.9–1000 μg/mL. This broad concentration range was used to define the cytotoxic and antiviral concentration–response curves required for calculation of the CC_50_, IC_50_, and SI. ENE exhibited a CC_50_ of 965.3 μg/mL and an IC_50_ of 29.61 μg/mL, resulting in an SI of 32.60 against A/PR8 (Figure 1A). Chloroquine exhibited a lower IC_50_ of 4.79 μM but also showed greater cytotoxicity, with a CC_50_ of 87.30 μM and an SI of 18.23 (Figure 1B). Because it is mathematically and pharmacologically inappropriate to directly compare the absolute antiviral potency (IC_50_) of a multi-component crude extract (measured in μg/mL) with a purified reference compound (measured in μM), we focused exclusively on the dimensionless SI to illustrate the favorable safety margin of ENE. Although the screening range extended to 1000 μg/mL to permit estimation of the complete concentration–response profile, clear antiviral activity was already evident within the range of ≤100 μg/mL, where A549 cell viability remained high.

To determine whether the antiviral activity could be reproduced when ENE exposure was restricted to a defined stage of infection, time-of-addition experiments were performed using separate pre-treatment, co-treatment, and post-treatment protocols. Pre-treatment of A549 cells with ENE did not produce a concentration-dependent reduction in A/PR8-induced cytopathic effects, and no inhibitory concentration could be calculated under this condition (Figure S1A,B). Although a small reduction in viral RNA was detected at an isolated high concentration, this change was not accompanied by a corresponding decrease in infectious progeny titers (Figure S1C,D). Similarly, co-treatment failed to produce consistent inhibition of virus-induced cytopathic effects, viral RNA abundance, or infectious progeny production (Figure S1E–H). These results indicated that exposure to ENE before or during viral inoculation alone was insufficient to produce a reproducible antiviral response. Because these time-of-addition assays empirically determined that the antiviral efficacy was exclusively restricted to the post-entry phase, it was methodologically necessary to transition to a strict post-treatment-only protocol for all downstream mechanistic evaluations to accurately reflect the extract’s therapeutic mode of action.

Based on the active concentration range identified in the preliminary screening, concentrations at or below 100 μg/mL were subsequently evaluated under the more restrictive post-treatment-only protocol, in which ENE was applied and continuously maintained for 48 h after viral adsorption (Figure 1C). Under these conditions, RT-qPCR analysis showed that ENE significantly reduced A/PR8 RNA levels beginning at 10 μg/mL, with progressively greater reductions at 25–100 μg/mL (Figure 1D). Infectious progeny titers showed a corresponding concentration-dependent decrease, becoming significantly reduced at 10 μg/mL and declining further at higher concentrations (Figure 1E).

Western blot analysis further demonstrated a concentration-dependent reduction in IAV nucleoprotein expression, with the most pronounced decrease observed following treatment with 50 μg/mL ENE (Figure 1F). Immunofluorescence analysis yielded a comparable pattern: treatment with 1 μg/mL ENE produced little apparent change in intracellular NP expression, whereas 10 and 50 μg/mL ENE progressively reduced the NP-associated fluorescence signal (Figure 1G). Quantitative image analysis confirmed significant reductions in viral protein expression at 10 and 50 μg/mL ENE (Figure 1H). These findings demonstrate that the antiviral activity identified during preliminary screening was retained at concentrations of ≤100 μg/mL under a continuous post-infection treatment condition.

### 3.2. ENE Attenuates A/PR8-Induced RIPK3–MLKL Necroptotic Signaling in A549 Cells

**Figure 2:**
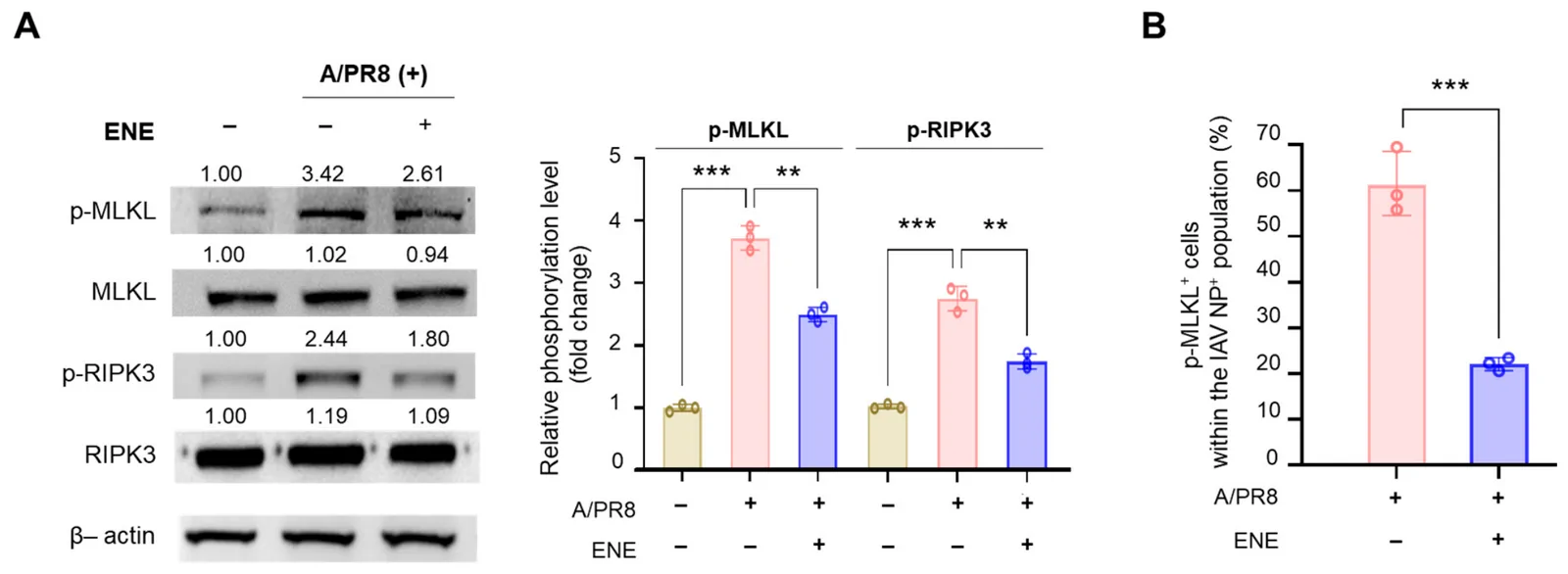
ENE attenuates A/PR8-induced RIPK3–MLKL necroptotic signaling in A549 cells. (**A**) Western blot analysis of phospho-RIPK3, total RIPK3, phospho-MLKL, and total MLKL in mock-infected vehicle-treated, A/PR8-infected vehicle-treated, and A/PR8-infected ENE-treated A549 cells. ENE was administered at 50 μg/mL, and β-actin was used as the loading control. Numbers above the target-protein bands indicate β-actin-normalized relative intensities. The adjacent graphs show the densitometric ratios of phospho-RIPK3 to total RIPK3 and phospho-MLKL to total MLKL. (**B**) Flow-cytometric quantification of p-MLKL-positive cells within the IAV NP-positive population. Data are presented as the mean ± SD of three independent experiments. ** indicates *p* < 0.01 and *** indicates *p* < 0.001 versus the A/PR8-infected vehicle-treated group.

To determine whether the post-infection antiviral activity of ENE was associated with modulation of necroptotic signaling, activation of the RIPK3–MLKL pathway was examined in A/PR8-infected A549 cells. Western blot analysis showed that A/PR8 infection increased the phosphorylation of RIPK3 at Ser227 and MLKL at Ser358 compared with the mock-infected, vehicle-treated control. Post-infection treatment with 50 μg/mL ENE reduced both p-RIPK3 and p-MLKL signals relative to those in the A/PR8-infected, vehicle-treated group (Figure 2A). Total RIPK3 and MLKL were analyzed in parallel to evaluate the phosphorylation status of the corresponding proteins. These findings indicate that ENE attenuated A/PR8-induced activation of the RIPK3–MLKL signaling axis.

MLKL phosphorylation was further evaluated specifically within the virus-infected cell population by two-color intracellular flow cytometry. A/PR8-infected cells were identified based on intracellular IAV NP expression, and the proportion of p-MLKL-positive cells within the NP-positive population was calculated by dividing the number of NP-positive/p-MLKL-positive double-positive events by the total number of NP-positive events and multiplying by 100. A/PR8 infection markedly increased the proportion of p-MLKL-positive cells within the NP-positive population, whereas ENE treatment significantly reduced this proportion (Figure 2B). Restricting the analysis to NP-positive events enabled MLKL phosphorylation to be assessed directly within the infected-cell population.

Taken together, the reductions in RIPK3 and MLKL phosphorylation detected by Western blotting, together with the decreased proportion of p-MLKL-positive cells among IAV-infected cells, indicate that ENE treatment is associated with the attenuation of A/PR8-associated RIPK3–MLKL necroptotic signaling in A549 cells.

### 3.3. Preliminary Screening and Post-Treatment Validation of ENE Against ZIKV and DENV2

Following the evaluation of ENE against A/PR8, its antiviral activity was examined against ZIKV and DENV2. Preliminary concentration–response screening was performed in Vero E6 cells using the combined pre- and post-infection treatment regimen over an ENE concentration range of 3.9–1000 μg/mL.

**Figure 3:**
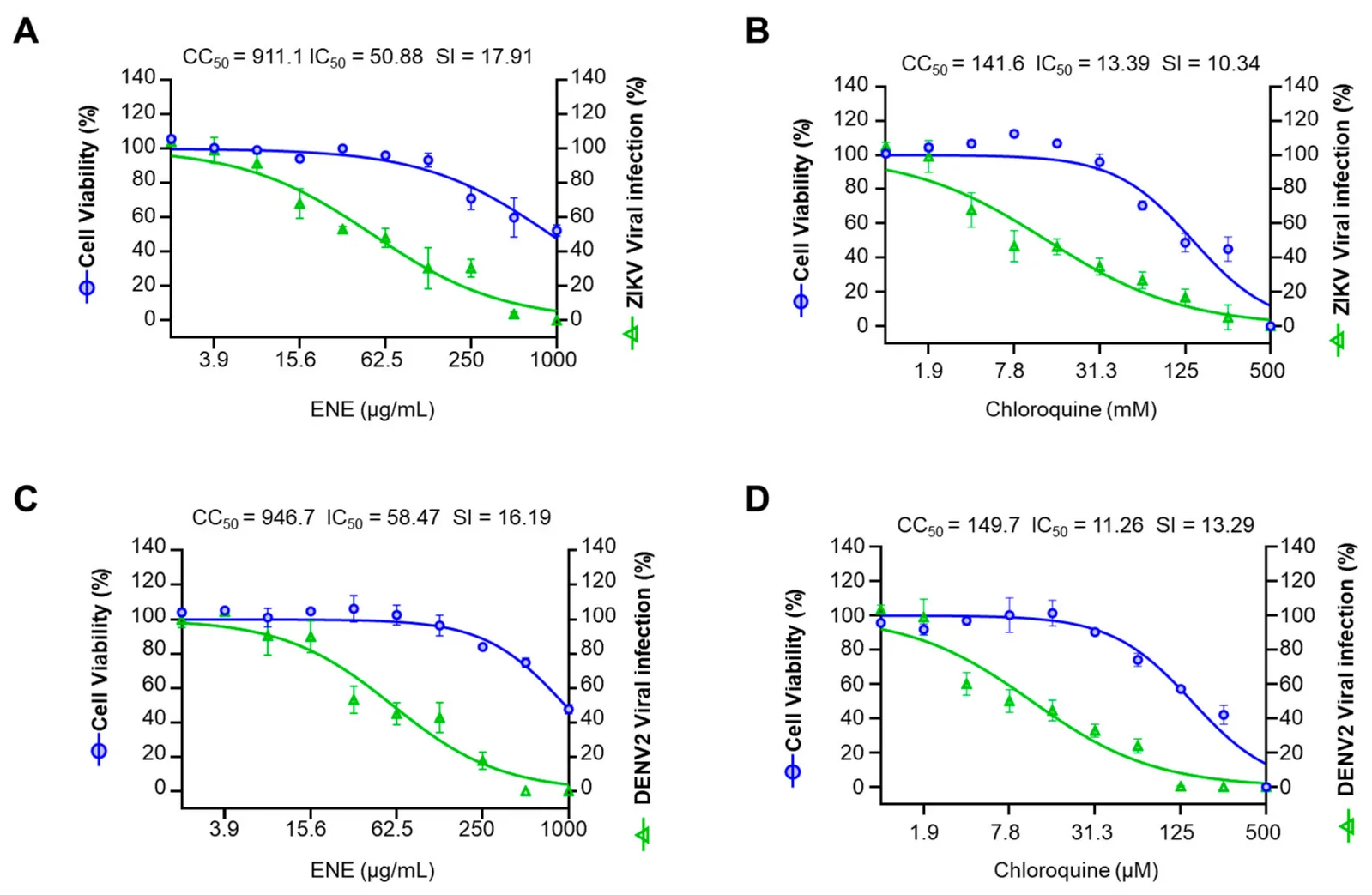
Preliminary concentration–response screening of ENE and chloroquine against ZIKV and DENV2 in Vero E6 cells. (**A**) Cytotoxicity and antiviral activity of ENE against ZIKV. (**B**) Cytotoxicity and antiviral activity of chloroquine against ZIKV. (**C**) Cytotoxicity and antiviral activity of ENE against DENV2. (**D**) Cytotoxicity and antiviral activity of chloroquine against DENV2. Blue curves represent cell viability, and green curves represent viral infectivity. Data are presented as the mean ± SD of three independent experiments. DENV2, dengue virus serotype 2; ZIKV, Zika virus.

Against ZIKV, ENE exhibited a CC_50_ of 911.1 μg/mL and an IC_50_ of 50.88 μg/mL, resulting in an SI of 17.91 (Figure 3A). Chloroquine showed greater apparent potency but also greater cytotoxicity, yielding an SI of 10.34 under the corresponding screening conditions (Figure 3B). ENE produced a similar concentration-dependent antiviral response against DENV2, with a CC_50_ of 946.7 μg/mL, an IC_50_ of 58.47 μg/mL, and an SI of 16.19 (Figure 3C). Chloroquine yielded an SI of 13.29 against DENV2 (Figure 3D). These findings demonstrated concentration-dependent antiviral activity of ENE against both flaviviruses under the preliminary screening regimen.

**Figure 4:**
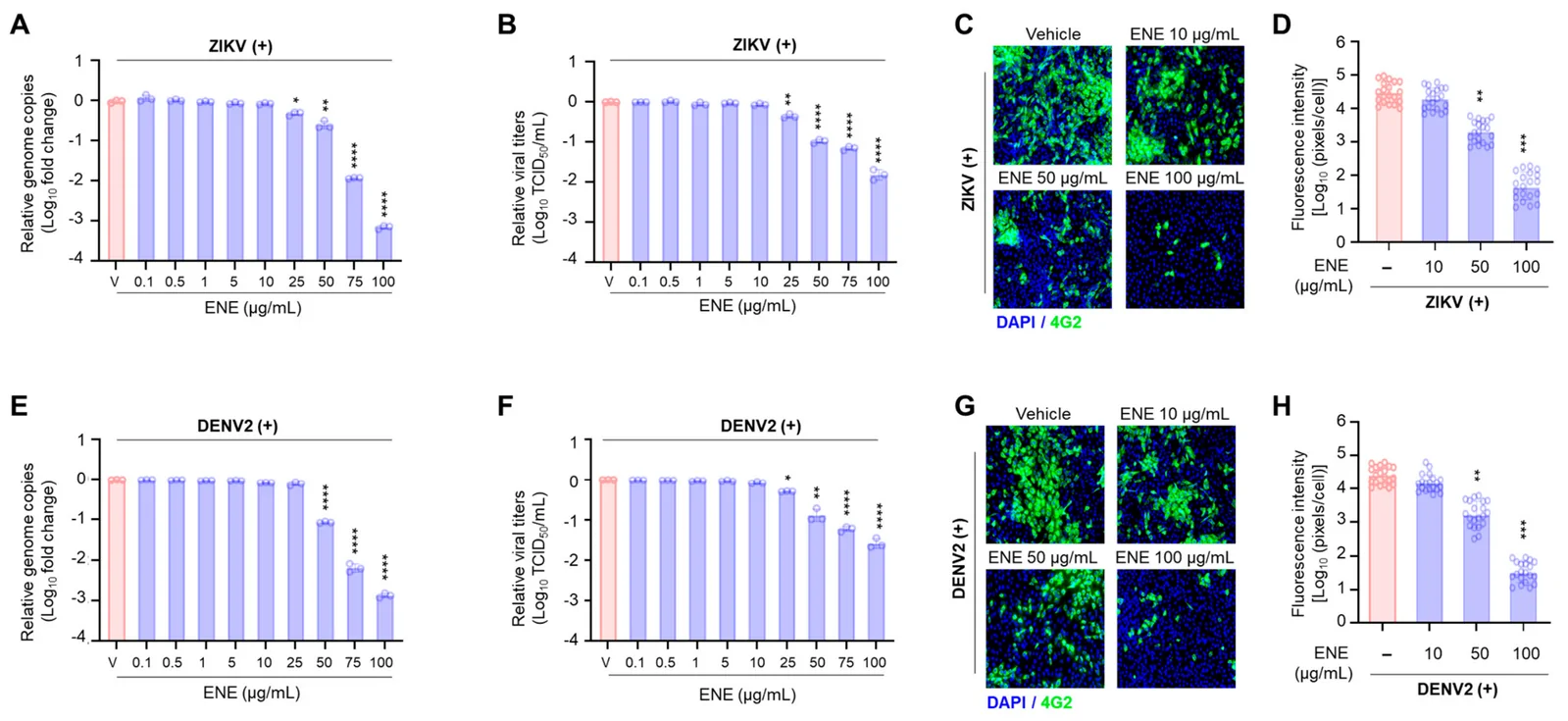
Post-treatment with ENE suppresses ZIKV and DENV2 replication in Vero E6 cells. (**A**–**D**) Effects of ENE on ZIKV infection. (**A**) Relative ZIKV genome copies (Log_10_ fold change) measured by RT-qPCR. (**B**) Relative infectious progeny titers (Log_10_ TCID_50_/mL) measured using the TCID_50_ assay. (**C**) Representative immunofluorescence images of ZIKV-infected cells treated with vehicle or ENE at 10, 50, or 100 μg/mL. Flavivirus envelope protein was detected using the 4G2 antibody, and nuclei were counterstained with DAPI. (**D**) Quantification of 4G2-associated fluorescence expressed as log_10_-transformed fluorescence-positive pixels per cell. (**E**–**H**) Effects of ENE on DENV2 infection. (**E**) Relative DENV2 genome copies (Log_10_ fold change) measured by RT-qPCR. (**F**) Relative infectious progeny titers (Log_10_ TCID_50_/mL) measured using the TCID_50_ assay. (**G**) Representative immunofluorescence images of DENV2-infected cells treated with vehicle or ENE at 10, 50, or 100 μg/mL. Viral envelope protein and nuclei were visualized using the 4G2 antibody and DAPI, respectively. (**H**) Quantification of 4G2-associated fluorescence expressed as log_10_-transformed fluorescence-positive pixels per cell. Quantitative data are presented as the mean ± SD of three independent experiments. *, *p* < 0.05; **, *p* < 0.01; ***, *p* < 0.001; and ****, *p* < 0.0001 versus the corresponding virus-infected vehicle-treated group.

As clear antiviral activity against both ZIKV and DENV2 was already evident within the ≤100 μg/mL range while Vero E6 cell viability remained high, subsequent virological assays were restricted to concentrations at or below 100 μg/mL and conducted using the post-treatment-only protocol. In ZIKV-infected cells, a continuous post-infection exposure to ENE for 48 h significantly reduced viral RNA levels at 25 μg/mL and higher with progressively greater reductions observed at 50–100 μg/mL (Figure 4A). Infectious progeny titers showed a corresponding concentration-dependent decline over the same active concentration range (Figure 4B). Immunofluorescence analysis further demonstrated a progressive loss of intracellular flavivirus envelope protein signals following treatment with 10, 50, and 100 μg/mL ENE (Figure 4C). Quantitative image analysis confirmed significant reductions in 4G2-associated fluorescence at 50 and 100 μg/mL (Figure 4D).

A comparable response was observed in DENV2-infected Vero E6 cells. Post-treatment with ENE significantly reduced viral RNA levels beginning at 25 μg/mL, with greater inhibition at 50–100 μg/mL (Figure 4E). Infectious progeny titers were similarly reduced in a concentration-dependent manner (Figure 4F). Consistent with these findings, intracellular 4G2 signals declined with increasing ENE concentrations (Figure 4G), and quantitative image analysis demonstrated significant reductions at 50 and 100 μg/mL (Figure 4H).

Together, these results demonstrate that the antiviral activity initially identified by broad-range preliminary screening was reproduced against both ZIKV and DENV2 under a continuous post-treatment-only condition using concentrations of ≤100 μg/mL. In conjunction with the corresponding A/PR8 findings, these results support a multi-virus in vitro antiviral profile of ENE and provided the rationale for examining virus-associated MLKL phosphorylation.

### 3.4. ENE Attenuates ZIKV- and DENV2-Associated MLKL Phosphorylation in Vero E6 Cells

Following the demonstration that ENE suppressed ZIKV and DENV2 genome accumulation, infectious progeny production, and viral antigen expression, we next examined whether these antiviral effects were accompanied by altered MLKL phosphorylation in flavivirus-infected cells. Two-color intracellular flow cytometry was used to identify flavivirus-infected cells based on 4G2-positive envelope protein staining and to quantify p-MLKL-positive events within the corresponding infected-cell population.

**Figure 5:**
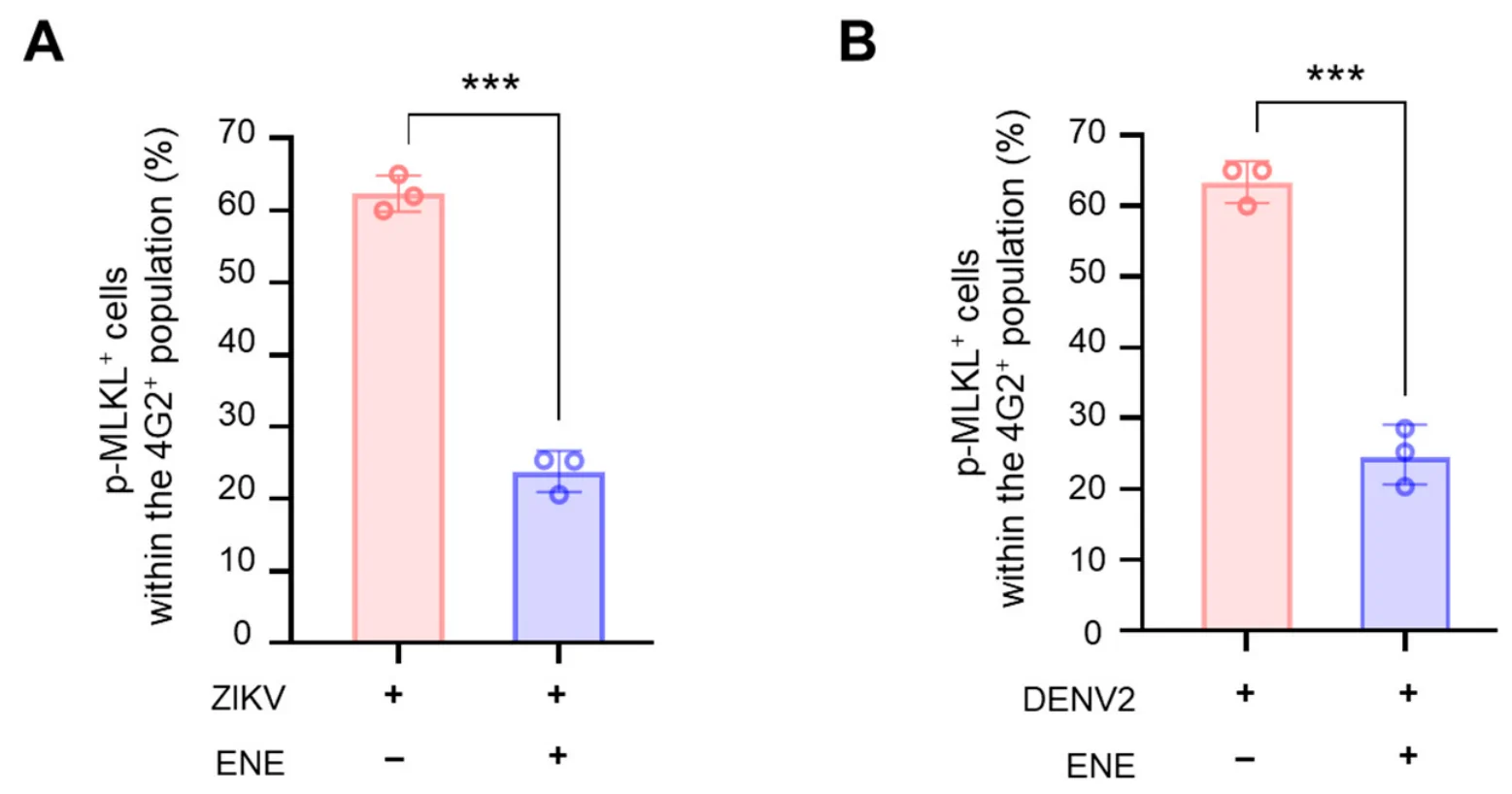
ENE reduces MLKL phosphorylation within ZIKV- and DENV2-infected Vero E6 cell populations. (**A**) Flow-cytometric quantification of p-MLKL-positive cells within the flavivirus 4G2-positive population in ZIKV-infected Vero E6 cells treated with vehicle or 100 μg/mL ENE. (**B**) Flow-cytometric quantification of p-MLKL-positive cells within the flavivirus 4G2-positive population in DENV2-infected Vero E6 cells treated with vehicle or 100 μg/mL ENE. Values represent p-MLKL-positive events expressed as a percentage of the total 4G2-positive population. Data are presented as the mean ± SD of three independent experiments. ***, *p* < 0.001 versus the corresponding virus-infected, vehicle-treated group.

In ZIKV-infected, vehicle-treated cultures, a substantial proportion of the 4G2-positive cell population was also positive for p-MLKL. Post-infection treatment with 100 μg/mL ENE significantly reduced the percentage of p-MLKL-positive cells within the ZIKV 4G2-positive population (Figure 5A). This result indicates that the reduction in ZIKV replication observed following ENE treatment was accompanied by attenuation of virus-associated MLKL phosphorylation.

A comparable response was observed in DENV2-infected Vero E6 cells. The proportion of p-MLKL-positive cells within the DENV2 4G2-positive population was markedly lower in ENE-treated cultures than in the corresponding virus-infected, vehicle-treated cultures (Figure 5B). Thus, the effect of ENE on MLKL phosphorylation was not restricted to ZIKV but was also observed during DENV2 infection.

Together with the reduction in RIPK3–MLKL phosphorylation observed in A/PR8-infected A549 cells, these findings show a consistent association between ENE treatment and attenuated virus-associated MLKL phosphorylation across influenza virus and flavivirus infection models. The results therefore support reduced MLKL-associated necroptotic signaling as a shared host-response feature accompanying the antiviral activity of ENE.

### 3.5. Preliminary Screening Demonstrates Antiviral Activity of ENE Against H3N2 and Influenza B Viruses

Finally, to determine whether the anti-influenza activity of ENE was restricted to the A/PR8 H1N1 strain, A/Wisconsin (H3N2) and B/Florida were evaluated in A549 cells using the same combined pre- and post-infection preliminary screening regimen.

**Figure 6:**
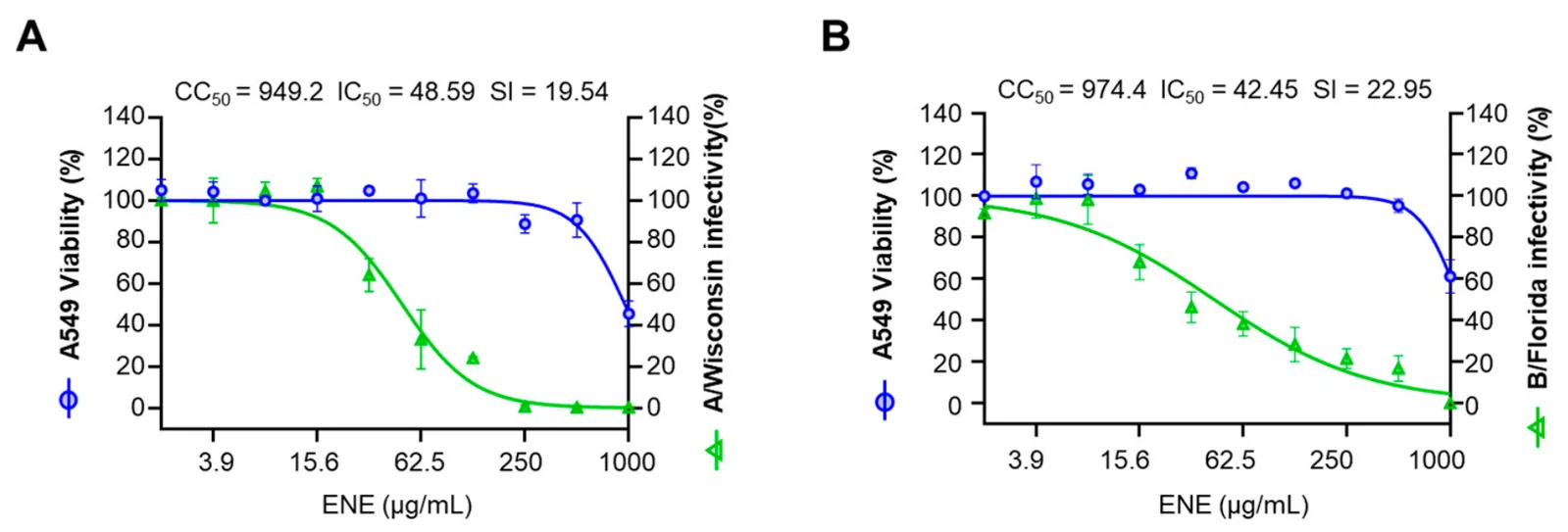
Preliminary screening demonstrates antiviral activity of ENE against H3N2 and influenza B viruses in A549 cells. (**A**) Cytotoxicity and antiviral activity of ENE against A/Wisconsin (H3N2). (**B**) Cytotoxicity and antiviral activity of ENE against B/Florida. Data are presented as the mean ± SD of three independent experiments.

ENE reduced A/Wisconsin-associated cytopathic effects in a concentration-dependent manner while maintaining high cell viability over a broad concentration range. The calculated CC_50_ was 949.2 μg/mL, whereas the IC_50_ was 48.59 μg/mL, resulting in an SI of 19.54 (Figure 6A). A similar concentration-dependent antiviral response was observed against B/Florida, for which ENE exhibited a CC_50_ of 974.4 μg/mL, an IC_50_ of 42.45 μg/mL, and an SI of 22.95 (Figure 6B).

These preliminary screening results indicate that the antiviral activity of ENE was not confined to the A/PR8 H1N1 strain but extended to an H3N2 influenza A virus and an influenza B virus. Together with the post-treatment validation obtained for A/PR8, ZIKV, and DENV2, these findings support a multi-virus in vitro antiviral profile for ENE across distinct enveloped RNA viruses.

## 4. Discussion

The present study identifies *Entodesma navicula* as a previously underexplored marine-animal source of antiviral bioactivity. ENE exhibited post-infection activity against influenza A and B viruses as well as ZIKV and DENV2, and this antiviral phenotype was consistently accompanied by reduced virus-associated MLKL phosphorylation. In the A/PR8 model, reduced MLKL phosphorylation was further supported by attenuation of RIPK3 phosphorylation, linking ENE treatment to modulation of the RIPK3–MLKL signaling axis. The principal significance of these findings therefore lies not only in the range of susceptible viruses but also in the convergence between antiviral activity and a regulated cell-death pathway relevant to viral pathogenesis.

The time-of-addition findings provide an important clue to the antiviral profile of ENE. Its lack of reproducible activity during pre- or co-treatment, together with its efficacy after viral adsorption, argues against cellular preconditioning, direct virion inactivation, or inhibition of attachment and entry as the predominant mechanism in the A/PR8 model. Instead, ENE is more likely to affect an intracellular event after entry, such as viral RNA synthesis, protein production, assembly, or a host dependency required during productive infection. However, because ENE is a multi-component crude extract, identifying the precise molecular target stage within the viral life cycle is not feasible at this juncture, necessitating future targeted molecular assays utilizing purified compounds. Furthermore, it should be noted that the continuous 48-h post-infection exposure to ENE, rather than a transient one-hour drug pulse, was necessary to observe this robust and long-lasting inhibitory effect. Additionally, the selection of an MOI of 0.1 for these post-treatment evaluations was an empirical decision based on preliminary optimizations to establish a reliable analytical window at 48 h; evaluating efficacy under varying MOI conditions remains necessary to fully define drug sensitivity dynamics. This profile differs from that of pyropheophorbide a isolated from the mussel *Musculus senhousei*, which inhibits influenza virus at an early stage by altering virion envelope lipids [20]. The activity of ENE across different influenza viruses and flaviviruses could reflect interference with a shared host-cell process, as demonstrated for other host-directed antiviral strategies, but a crude extract may also contain multiple constituents acting through distinct mechanisms [25]. Because ENE is currently an uncharacterized crude extract without defined chemical structures, performing in silico computational simulations (e.g., molecular docking) to explore these host–pathogen interactions is not yet feasible, but is strategically planned once specific active compounds are structurally identified. Because time-of-addition analysis was performed only with A/PR8, the same post-entry mechanism should not yet be assumed for ZIKV or DENV2.

The reduction in RIPK3 and MLKL phosphorylation during A/PR8 infection must be interpreted in the context of the complex role of necroptosis in influenza pathogenesis. Replicating IAV generates RNA structures recognized by ZBP1, leading to RIPK3 activation and downstream MLKL phosphorylation [8,9,10]. Viral proteins may also directly influence this pathway; IAV NS1 has been reported to interact with MLKL and promote its oligomerization and membrane translocation [26]. The present Western blot and infected-cell-restricted flow-cytometric results are consistent with attenuation of this virus-associated signaling response by ENE. However, RIPK3–MLKL signaling is not uniformly proviral. Necroptosis can eliminate infected cells and function as an effective antiviral defense during IAV infection, particularly when alternative cell-death routes are impaired [7,27]. Consequently, the current data do not establish that inhibition of necroptosis is the direct cause of reduced viral replication. ENE may primarily inhibit viral replication, thereby reducing the viral RNA and protein signals that activate RIPK3–MLKL signaling, or it may simultaneously limit viral propagation and excessive lytic inflammatory responses. Therefore, our findings indicate an association between ENE treatment and attenuated necroptosis-associated signaling, rather than definitively establishing direct causality or functional inhibition of terminal necroptotic cell death. Determining the exact causal sequence—whether the reduction in viral replication precedes the attenuation of necroptosis, or whether the modulation of host-cell death actively limits viral propagation and protects cells—requires functional genetic interventions (e.g., RIPK3 or MLKL knockouts/knockdowns), which is a limitation of the current study. Resolving the direction of this relationship will require time-resolved and genetic experiments.

The flavivirus findings further emphasize the context dependence of RIPK3–MLKL biology. ZIKV-induced RIPK3-dependent necroptosis restricts viral replication in human astrocytes, indicating that this response can be protective in susceptible neural cells [11]. RIPK3 can also induce an antiviral metabolic state independently of terminal necroptotic death, demonstrating that changes in RIPK3-related signaling cannot always be interpreted solely as changes in cell death [28]. Conversely, DENV can target RIPK1 and suppress receptor-induced inflammatory and MLKL-associated signaling in human myeloid cells [12]. The reduction in p-MLKL-positive cells within the 4G2-positive populations therefore indicates that ENE altered MLKL activation within ZIKV- and DENV2-infected Vero E6 cells, but it does not define whether this alteration was upstream or downstream of viral inhibition. Moreover, Vero cells contain a homozygous genomic deletion encompassing the type I interferon gene cluster, which limits extrapolation of these findings to immunocompetent vertebrate cells [29]. We acknowledge this genomic deletion as a limitation; however, the basal necroptosis machinery, including endogenous RIPK3 and MLKL, remains functionally intact in Vero E6 cells. Recent virological studies demonstrate that necroptotic cell death can be robustly triggered in Vero E6 cells through IFN-independent pathways during RNA virus infections [30,31]. While necroptotic signaling is operational and measurable, interpreting broad innate immune responses in an IFN-deficient system requires extreme caution.

The marine-bivalve origin of ENE adds a distinct natural-product dimension to the study. Antiviral activity has previously been reported in crude extracts from mussels, oysters, and clams, including activity against influenza A and B viruses [14] and herpesviruses [15]. Antiviral molecules characterized from bivalves include oyster hemolymph components [18], the Pacific oyster hemolymph protein cavortin [17], the mussel-derived peptide myticin C [16], and pyropheophorbide a from *M. senhousei* [20]. Based on previous literature investigating marine bivalves, the antiviral activity may originate from conserved marine metabolites such as bioactive peptides [16] or specific lipid derivatives [20]. In contrast, previous work on *E. navicula* has largely addressed shell structure and biomineralization rather than pharmacological activity [19]. ENE therefore expands the taxonomic range of bivalves considered for antiviral discovery. Nevertheless, because a whole-specimen 70% ethanolic crude extract was tested, the activity cannot yet be assigned to a defined endogenous bivalve molecule. At this preliminary screening stage, comprehensive chemical profiling (e.g., HPLC/LC-MS) has not been performed, which is a significant limitation. Additionally, because ENE is a crude extract, relatively high mass concentrations are required to achieve antiviral efficacy, and acknowledging these differences in chemical composition is necessary when comparing it to a purified reference compound. However, evaluating crude extracts at these concentrations is a widely accepted characteristic in natural product screening, and the IC_50_ values of ENE (29–58 μg/mL) fall well within the clinically relevant threshold (<100 μg/mL) for promising anti-infective potential [32]. Bioactivity-guided fractionation, high-resolution mass spectrometry, and structural identification will be required to determine whether one constituent accounts for the cross-virus activity or whether several compounds contribute independently.

Several mechanistic limitations should also be considered. MLKL phosphorylation is a central activation event, but completed necroptosis additionally requires conformational activation, oligomerization, membrane translocation, and loss of membrane integrity [4,5,33,34]. Accordingly, the present results establish attenuation of necroptosis-associated signaling rather than direct measurement of completed necroptotic death. Confirmation should include MLKL oligomerization or membrane translocation, membrane-impermeant dye uptake, lactate dehydrogenase release, and genetic depletion of RIPK3 or MLKL. RIPK3 activation should also be evaluated in the flavivirus models, together with confirmation of total MLKL expression and antibody cross-reactivity in Vero E6 cells. The absence of population-level total MLKL Western blot data and orthogonal microscopic validation of flow cytometry in Vero E6 cells represents a methodological limitation, though our flow cytometry specifically provided single-cell resolution within the infected subpopulation. Furthermore, relying on a single late time point (48 h) and single concentrations restricts a comprehensive understanding of dynamic signaling kinetics. Other technical limitations include the need for orthogonal, non-metabolic assays (e.g., ATP-based) to definitively validate CC_50_ values against latent colorimetric MTT interference, the potential influence of subtle morphological changes on pixel-based immunofluorescence quantification, and the omission of virus-specific antiviral drugs (e.g., Oseltamivir) as positive controls in mechanistic assays for benchmarking absolute potency. These experiments would determine whether RIPK3–MLKL signaling is required for ENE-mediated antiviral activity or instead declines as a consequence of reduced viral burden.

The use of A549 and Vero E6 cells further limits direct translational interpretation, and the multi-virus antiviral profile observed here remains an in vitro finding across a defined virus panel, indicating that ENE is effective against multiple influenza viruses and flaviviruses, rather than evidence of universal cross-family antiviral activity. Validation in primary airway epithelial cells, flavivirus-relevant endothelial or neural cells, organoid systems, and immunocompetent animal models will be required. In parallel, standardized extract production, batch reproducibility, pharmacokinetics, bioavailability, and systemic toxicity must be established before post-exposure efficacy can be interpreted as therapeutic potential. Despite these limitations, the study connects marine-animal bioprospecting with host–pathogen interactions and regulated inflammatory cell death, themes directly relevant to veterinary virology and One Health research. Overall, ENE represents a promising source for antiviral lead discovery, while the observed reduction in RIPK3–MLKL signaling should presently be regarded as a mechanistically relevant association that warrants causal validation.

## 5. Conclusions

This study identifies *Entodesma navicula* as a previously underexplored marine-animal source of post-infection antiviral bioactivity against influenza viruses and flaviviruses. The consistent association between ENE-mediated viral suppression and reduced RIPK3–MLKL signaling suggests that bivalve-derived constituents may act at the interface of viral replication and regulated host-cell death, although a causal role for necroptosis remains to be established. These findings broaden the range of marine bioresources considered for antiviral discovery and support ENE as a basis for bioactivity-guided fractionation and mechanistic investigation. Identification of the active constituents, followed by validation in primary-cell and animal models and assessment of safety and post-exposure efficacy, will be essential to determine its potential for veterinary antiviral development and One Health preparedness.

## Data Availability

The data supporting the conclusions of this article are included within the article. Raw data are available from the corresponding author upon reasonable request.

## Supplementary Materials

The following supporting information can be downloaded at https://www.mdpi.com/article/10.3390/ani16203226/s1. Figure S1: ENE does not produce consistent antiviral activity against A/PR8 under pre-treatment or co-treatment conditions. (A–D) Evaluation of ENE using the pre-treatment protocol. (A) Schematic representation of cell preparation, ENE exposure, A/PR8 inoculation, medium replacement, and sample collection. (B) Cell viability and A/PR8 infectivity assessed using an MTT-based cytopathic-effect reduction assay. CC_50_ and IC_50_ values were not determined because a concentration-dependent response was not observed. (C) Relative A/PR8 genome copies measured by RT-qPCR. (D) Relative infectious progeny titers measured by the 50% tissue culture infectious dose (TCID_50_) assay. (E–H) Evaluation of ENE using the co-treatment protocol. (E) Schematic representation of simultaneous A/PR8 and ENE inoculation, medium replacement, and sample collection. (F) Cell viability and A/PR8 infectivity assessed using an MTT-based cytopathic-effect reduction assay. CC_50_ and IC_50_ values were not determined because a concentration-dependent response was not observed. (G) Relative A/PR8 genome copies measured by RT-qPCR. (H) Relative infectious progeny titers measured by the TCID_50_ assay. Data are presented as the mean ± SD of three independent experiments. *, *p* < 0.05 versus the A/PR8-infected vehicle-control group. ENE, *Entodesma navicula* extract; N.D., not determined.
