# Supplementary material for: A Marine Bivalve (Entodesma navicula) Extract Suppresses the Replication of Influenza Viruses and Flaviviruses in Association with Reduced MLKL Phosphorylation
Source: Animals (Basel). 2026 Oct 14;16(20):3226. doi: 10.3390/ani16203226 (PMC13700874; doi:10.3390/ani16203226)

Supplementary Materials

Supplementary Figures

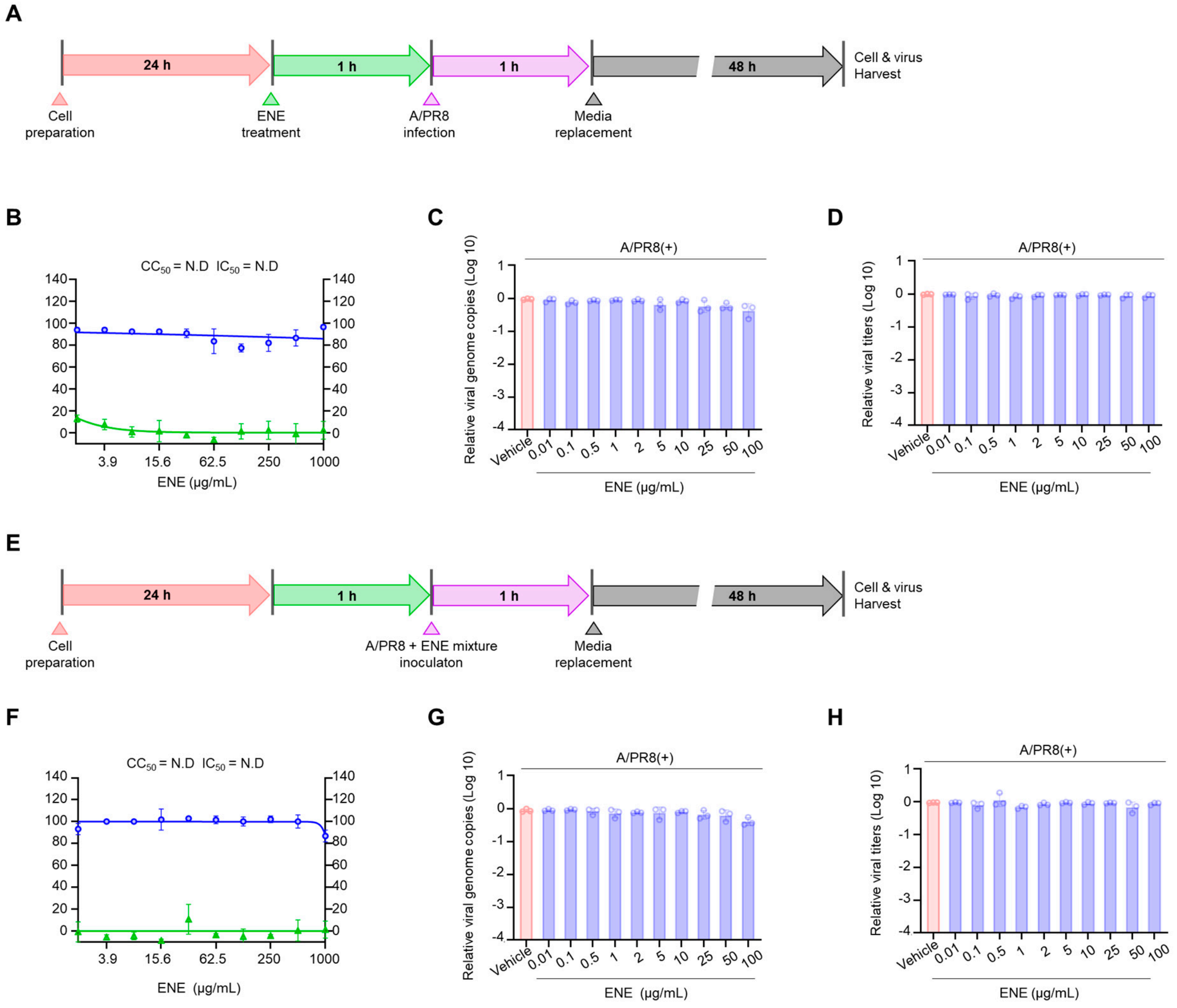

Supplementary Figure S1. ENE does not produce consistent antiviral activity against A/PR8 under pre-treatment or co-treatment conditions. (**A–D**) Evaluation of ENE using the pre-treatment protocol. (**A**) Schematic representation of cell preparation, ENE exposure, A/PR8 inoculation, medium replacement, and sample collection. (**B**) Cell viability and A/PR8 infectivity assessed using an MTT-based cytopathic-effect reduction assay. CC_{50} and IC_{50} values were not determined because a concentration-dependent response was not observed. (**C**) Relative A/PR8 genome copies measured by RT-qPCR. (**D**) Relative infectious progeny titers measured by the 50% tissue culture infectious dose ($TCID_{50}$) assay. (**E–H**) Evaluation of ENE using the co-treatment protocol. (**E**) Schematic representation of simultaneous A/PR8 and ENE inoculation, medium replacement, and sample collection. (**F**) Cell viability and A/PR8 infectivity assessed using an MTT-based cytopathic-effect reduction assay. CC_{50} and IC_{50} values were not determined because a concentration-dependent response was not observed. (**G**) Relative A/PR8 genome copies measured by RT-qPCR. (**H**) Relative infectious progeny titers measured by the $TCID_{50}$ assay. Data are presented as the mean ± SD of three independent experiments. *, $p < 0.05$ versus the A/PR8-infected vehicle-control group. ENE, *Entodesma navicula* extract; N.D., not determined.

Western Blot Original Images

Blot image corresponding to Figure 1F

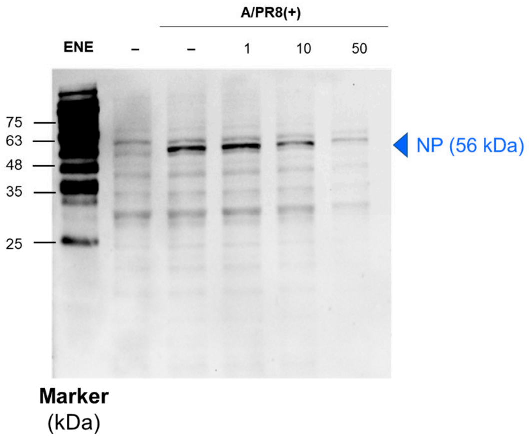

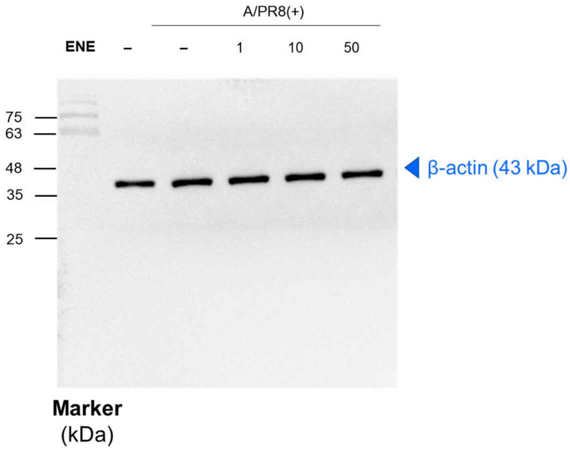

Blot image corresponding to Figure 2A

Original membrane for p-MLKL / Original image for Figure 2A

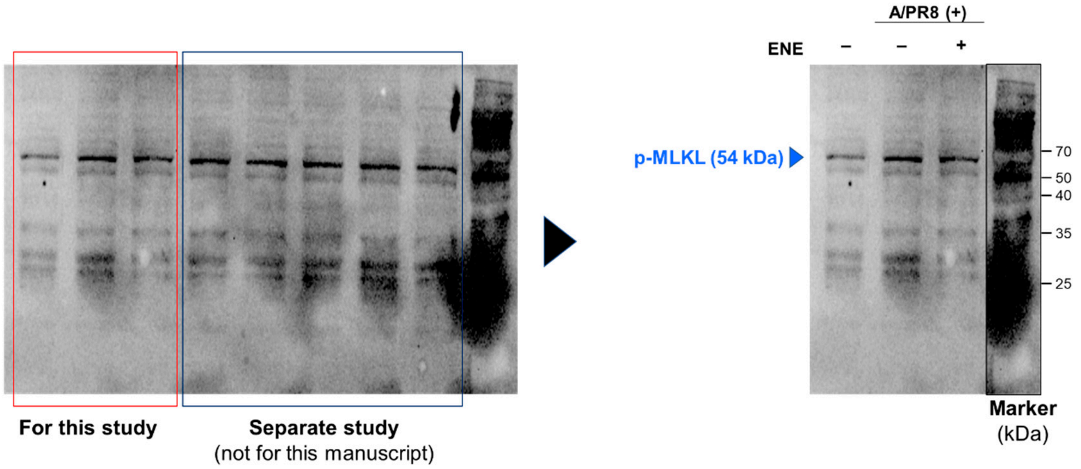

Original membrane for MLKL / Original image for Figure 2A

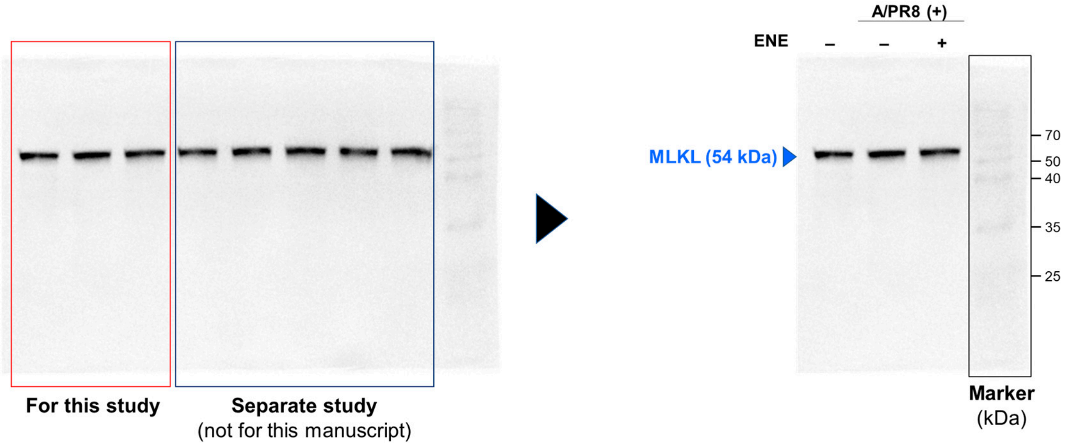

Original membrane for p-RIP3 / Original image for Figure 2A

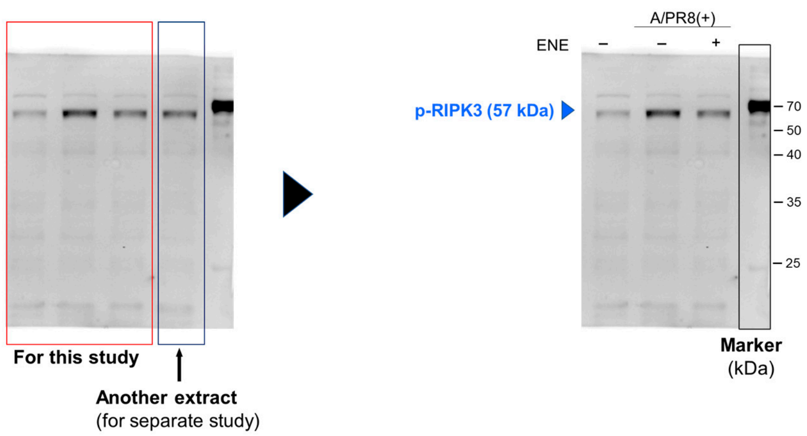

Original membrane for RIP3

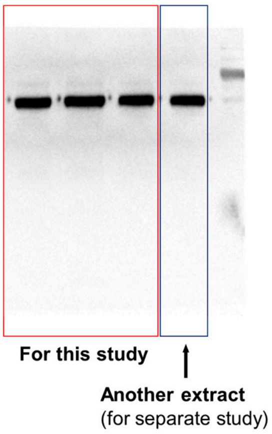

Original image for Figure 2A

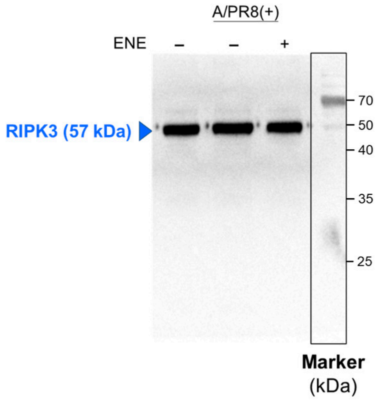

Original membrane for β-actin

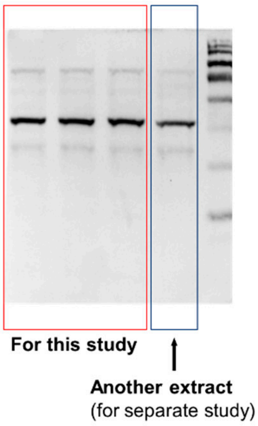

Original image for Figure 2A

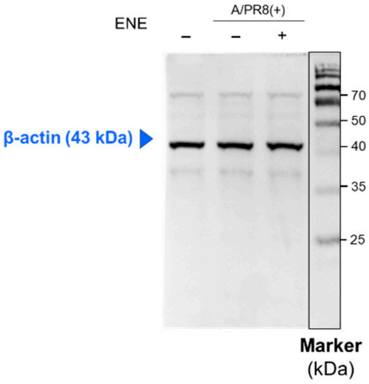

Supplement: Supplementary file 1 [file animals-16-03226-s001.zip › animals-4540674-supplementary.pdf]
